# RETRACTION: The effect of marathon on mRNA expression of anti-apoptotic and pro-apoptotic proteins and sirtuins family in male recreational long-distance runners

**DOI:** 10.1186/1472-6793-13-13

**Published:** 2014-01-10

**Authors:** Gabriella Marfe, Marco Tafani, Bruna Pucci, Carla Di Stefano, Manuela Indelicato, Angela Andreoli, Matteo Antonio Russo, Paola Sinibaldi-Salimei, Vincenzo Manzi

**Affiliations:** 1 Department of Experimental Medicine and Biochemical Sciences, University of Rome "Tor Vergata" Via Montpellier 1, 00133, Rome, Italy; 2 Department of Cellular and Molecular Pathology, IRCCS San Raffaele Pisana, Via dei Bonacolsi snc, 00163, Rome, Italy; 3 Human Nutrition Unit, University of Rome "Tor Vergata" Via Montpellier 1, 00133, Rome, Italy; 4 Department of Experimental Medicine, La Sapienza University, Viale Regina Elena 324, 00161, Rome, Italy; 5 School of Sport and Exercise Sciences, University of Rome Tor Vergata" Via Columbia s.n.c., 00133, Rome, Italy

## 

The authors have retracted the article [1]. Following publication of the article irregularities within the Northern blot figures were brought to the attention of the editors and subsequently confirmed by an investigation by the University of Rome Tor Vergata. We apologize to all affected parties.
